# Genome-wide association study of partial resistance to sclerotinia stem rot of cultivated soybean based on the detached leaf method

**DOI:** 10.1371/journal.pone.0233366

**Published:** 2020-05-18

**Authors:** Mingming Sun, Yan Jing, Xue Zhao, Weili Teng, Lijuan Qiu, Hongkun Zheng, Wenbin Li, Yingpeng Han

**Affiliations:** 1 Key Laboratory of Soybean Biology in Chinese Ministry of Education (key Laboratory of Soybean Biology and Breeding/Genetics of Chinese Agriculture Ministry), Northeast Agricultural University, Harbin, China; 2 Heilongjiang Journal Press of Agricultural Science and Technology, Heilongjiang Academy of Agricultural Sciences, Harbin, China; 3 Institute of Crop Science, National Key Facility for Crop Gene Resources and Genetic Improvement (NFCRI) Chinese Academy of Agricultural Sciences, Beijing, China; 4 Bioinformatics Division, Biomarker Technologies Corporation, Beijing, China; University of Guelph, CANADA

## Abstract

Sclerotinia stem rot (SSR) is a devastating fungal disease that causes severe yield losses of soybean worldwide. In the present study, a representative population of 185 soybean accessions was selected and utilized to identify the quantitative trait nucleotide (QTN) of partial resistance to soybean SSR *via* a genome-wide association study (GWAS). A total of 22,048 single-nucleotide polymorphisms (SNPs) with minor allele frequencies (MAF) > 5% and missing data < 3% were used to assess linkage disequilibrium (LD) levels. Association signals associated with SSR partial resistance were identified by two models, including compressed mixed linear model (CMLM) and multi-locus random-SNP-effect mixed linear model (mrMLM). Finally, seven QTNs with major effects (a known locus and six novel loci) *via* CMLM and nine novel QTNs with minor effects *via* mrMLM were detected in relation to partial resistance to SSR, respectively. One of all the novel loci (Gm05:14834789 on Chr.05), which was co-located by these two methods, might be a stable one that showed high significance in SSR partial resistance. Additionally, a total of 71 major and 85 minor candidate genes located in the 200-kb genomic region of each peak SNP detected by CMLM and mrMLM were found, respectively. By using a gene-based association, a total of six SNPs from three major effects genes and eight SNPs from four minor effects genes were identified. Of them, *Glyma*.*18G012200* has been characterized as a significant element in controlling fungal disease in plants.

## Introduction

As one of the most devastating and economically important fungal phytopathogens in soybean [*Glycine max* (L.) Merr.], sclerotinia stem rot (SSR) is caused by *Sclerotinia sclerotiorum* (Lib.) de Bary, and humidity and temperature have a profound impact on the prevalence, incidence, development and severity of this disease [1]. *Sclerotinia sclerotiorum* (Lib.) de Bary was recognized as an omnivorous plant pathogen with a broad host range and worldwide distribution. SSR was further expanded for rotation between soybean and other host plants. SSR could cause serious yield loss of soybean, especially in North America and northeastern China [2,3]. Agronomic management practices, including disease-free seeds, early planting dates, soil tillage, and adjusting row width and population density, contributed to the control of SSR severity; however, the effectiveness of these agronomic practices could be limited [4]. The resistant cultivar was the most economical and long-lasting means of managing this disease. Although no complete resistance to SSR was reported in soybean, different susceptibilities or partial resistance were still effective in controlling this disease [5]. The identification of cultivars with partial resistance may provide useful information for breeding new cultivar resistance to this disease.

Partial resistance to soybean SSR is a heritable quantitative trait [6,7], which is controlled by genes with main or minor effects and affected by environment and genotype by environment interactions [5]. Researchers have found that *Sclerotinia sclerotiorum*–soybean molecular interaction was a very complex process, involving many pathogen-released proteins [8] and other factors, for instance, oxalic acid, secreted effectors and cell wall degrading enzymes for the host to deal with [9–13]. Pan et al (2018) identified that SsSm1 functioned as an elicitor involved in hyphal development of *Sclerotinia sclerotiorum*, and silencing *SsSm1* could reduce the pathogenicity [14]. For the purpose of finding more functional genes, QTL analysis was conducted, based on a linkage mapping strategy and the bi-parent population, to position DNA markers associated with SSR resistance. At present, a total of 103 QTLs, distributed on 18 of all 20 soybean chromosomes, were reported in the Soybase databank (www.soybase.org). Among these QTLs, most were not reported again in other studies. Therefore, these identified QTLs were difficult to directly utilize in marker-assisted breeding (MAS) of SSR. Different from the bi-parent population of linkage analysis, genome-wide association analysis (GWAS) using a natural population of non-cross-derived lines could provide extensive recombination and shorter LD segments and could thus increase the resolution of marker-phenotype associations [15].

Recently, high-throughput sequencing and gene chip technology have further accelerated the development of GWAS, and GWAS has been a promising strategy for the identification of loci for genes of interest traits. Some studies have been reported for soybean SSR based on GWAS and highlighted its effectiveness aiming at improving the enhanced resistance to SSR. Wei et al. (2017) used two models, CMLM and FarmCPU, and finally found 125 genes around three loci with high confidence [10]. Four QTNs, located on chromosome 01 (Chr.01), Chr.15, Chr.19 and Chr.20, were detected through 130 tested samples and 7,864 single nucleotide polymorphisms (SNPs) based on the cotton pad method [16]. A total of 58 significant main effect QTNs and 24 epistatic QTNs for the partial resistance of SSR were finally identified on the basis of the phenotypes of 466 diverse soybean accessions obtaining from the cut stem method, and the genotyping data of 35,683 SNPs [1]. Zhao et al. (2015) identified five genomic regions that were significantly associated with stem pigmentation content after treatment with oxaloacetic acid, an indirect trait of SSR resistance in soybean [5]. To date, soybean cultivars from northeastern China have not evaluated SSR resistance based on the direct evaluation method.

In the present study, a total of 185 cultivars were evaluated for their partial resistance to SSR using the detached leaf method, and GWAS was performed based on 22,048 SNPs and these tested samples. The aim of this study was to screen resistance sources from Northeast China, to identify QTN associated with partial resistance of SSR, and to predict candidate genes located in QTN regions.

## Materials and methods

### Plant materials and phenotypic evaluation

A total of 185 Chinese representative accessions, including 38 landraces and 147 elite cultivars, were collected from northeastern China. Phenotypic evaluations of these tested accessions were conducted using the ‘detached leaf method’ described by Kull et al [17]. Five seeds of each tested soybean were germinated in 15-cm clay pots containing an equal mixture of soil (peat: soil: sand: vermiculite) and grown in a greenhouse (27 ± 1°C) under 16-h day length according to the detached leaf method described by Kull et al [17]. And experimental designs with a randomized complete block with three replications were utilized. The fully expanded leaves of 4-week-old soybeans were cut from the stem and then placed in a labeled, moistened paper towel. An 8-mm^2^ plug was placed fungus-side down centered on one side of the middle trifoliolate leaf between the main leaf vein and the leaf edge and gently pressed to ensure good contact with the leaf surface. After 48 h, the diameter of lesions among inoculated leaves was measured on the following 5 days and used as the phenotypic value.

### SNP genotyping data collection

The genomic DNA of the tested materials was isolated and then genotyped based on specific locus amplified fragment sequencing (SLAF-seq) methodology [18]. Two digest enzymes, Mse I (EC 3.1.21.4) and HaeIII (EC: 3.1.21.4) (Thermo Fisher Scientific Inc., Waltham, MA, USA.), were selected to produce more than 50,000 sequencing tags (approximately 300–500 bp in length) of each tested sample based on preliminary analysis of the reference genome, which was distributed in unique genomic regions of 20 soybean chromosomes. The sequencing libraries of each tested sample were defined by sequencing tags with the same genomic region. The barcode method with the Illumina Genome Analyzer II system (Illumina Inc., San Diego, CA, USA) was used to generate reads measuring 45 bp in length at both ends of sequencing tags from each accession library. The raw paired-end reads were aligned to the soybean reference genome using Short Oligonucleotide Alignment Program 2 (SOAP2) software. The raw reads in the same genomic position were used to define the SLAF groups *via* more than 58,000 high-quality SLAF tags from each tested sample. The SNPs were defined based on MAF > 0.05. The genotype was regarded as heterozygous when the depth of minor allele/ the total depth of the sample was ≥ 1/3.

Among the 185 soybean accessions, we picked on twenty-six lines with extreme phenotypic values for SSR, of which were thirteen resistant and thirteen susceptible lines, with a lower or higher level on the lesion length, respectively. A genome resequencing with 10-fold in depth was conducted using an Illumina HiSeq 2500 sequencer for the selected lines. Paired-end resequencing reads were mapped to the soybean Williams 82 reference genome (Version: Glyma.Wm82. a2) with BWA (Version: 0.6.1-r104) using the default parameters [19]. SAMtools48 (Version: 0.1.18) software was used in converting mapping results into the BAM format and filtering the unmapped and non-unique reads. Duplicated reads were filtered with the Picard package (picard.sourceforge.net, Version: 1.87). The BEDtools (Version: 2.17.0) coverage Bed program was used for computing the coverage of sequence alignments. A sequence was defined as absent when coverage was lower than 90% and present when coverage was higher than 90%. SNP detection was conducted by the Genome Analysis Toolkit (GATK, version 2.4-7-g5e89f01) and SAMtools [19]. Only the SNPs that were detected by both methods could be further analyzed. The SNPs with allele frequencies lower than 5% in the population were discarded. SNP annotations were performed based on the soybean genome (Version:Glyma.Wm82.a2) by the package ANNOVAR (Version: 2013-08-23) [19].

### Population structure evaluation and linkage disequilibrium (LD) analysis

The population structure of the association panel was analyzed through the principle component analysis (PCA) approach of GAPIT software [20]. LD between pairs of SNPs was defined based on the SNP threshold value (MAF> 0.05 and missing data < 3%) and R^2^ (squared allele frequency correlations) using the software TASSEL version 3.0 [21]. In contrast to the GWAS, missing SNP genotypes were not imputed with the major allele before LD analysis. Parameters in the program included MAF (> 0.05) and the integrity of each SNP (> 80%).

### Association mapping

Compressed mixed linear model (CMLM) in GAPIT [20] and multi-locus random-SNP-effect mixed linear model (mrMLM) were used to detect the association signals of partial resistance to SSR with major or minor effects, respectively, through 22,048 SNPs and 185 tested accessions. The significance thresholds for the association between SNP and traits were determined by -log10(*P*) ≥ 3 for CMLM model [22,23] and LOD≥ 3 for mrMLM model [24,25].

### Prediction of candidate genes controlling resistance to SSR

Candidate genes, located in the 200-kb genomic region of each peak SNP, were classified and then annotated with the soybean reference genome Williams 82 (Wm82. a2. v1, http://www.soybase.org) according to Cheng et al [26]. The SNP variations that occurred in the regions of candidate genes, including exonic regions, splicing sites, 5′UTRs and 3′UTRs, intronic regions, upstream and downstream regions, were detected based on genome re-sequencing data and analyzed using the compressed mixed linear model (CMLM) method to identify SSR-related haplotypes [21]. Significant SNPs affecting the target trait were claimed when the test statistics reached *p* < 0.01. Bio-Analytic Resource for Plant Biology database (BAR, http://bar.utoronto.ca/efp/cgi-bin/efpWeb.cgi) was used to further screen candidate genes for the resistance.

## Results

### Phenotypic characteristic of partial resistance to SSR in the association panel

**Fig 1:**
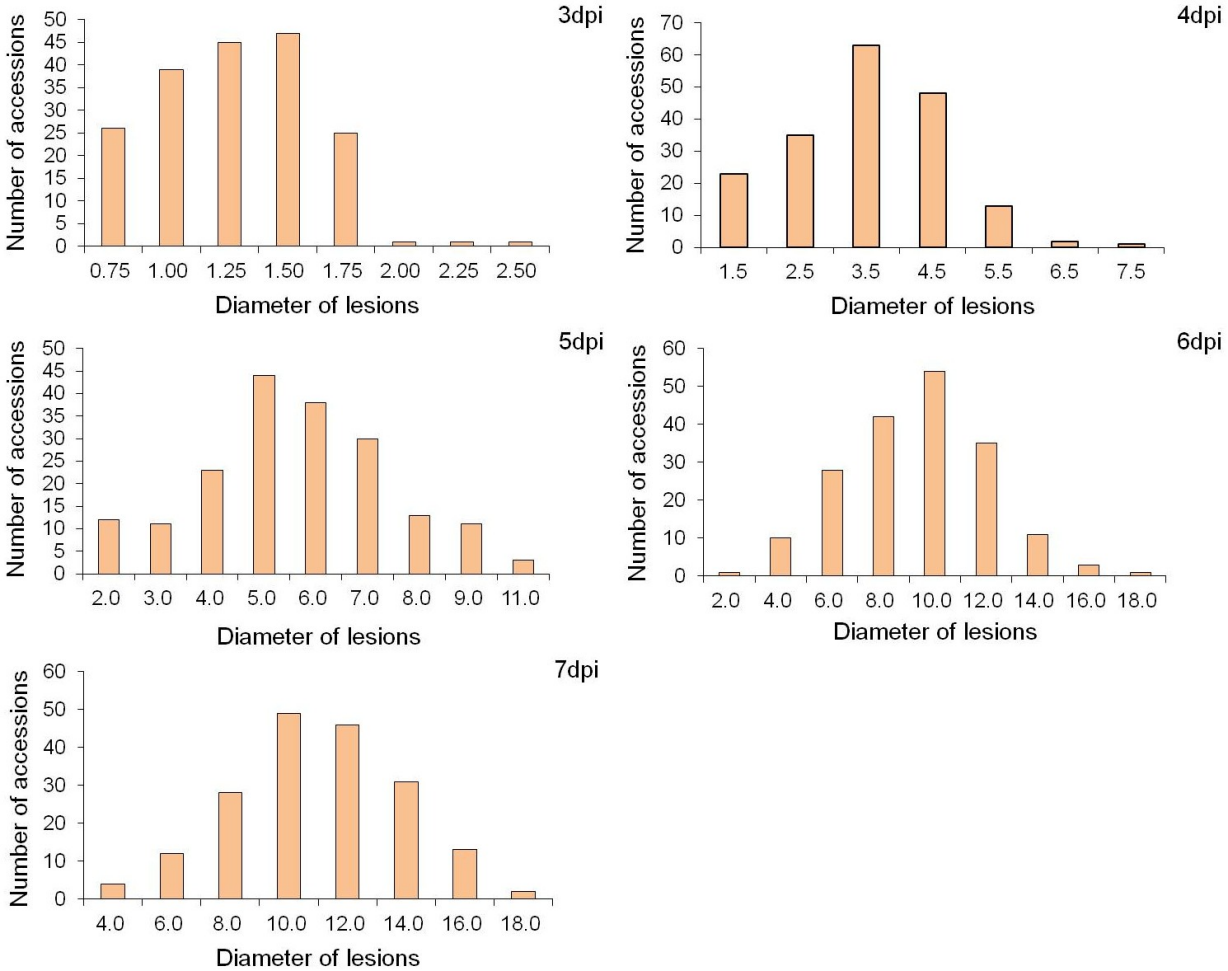
Distribution of partial resistance to sclerotinia stem rot (SSR) among 185 soybean accessions under five tested time points (‘3d’, ‘4d’, ‘5d’, ‘6d’, ‘7d’).

A wide range of resistance responses existed among the 185 samples after inoculation with SSR (S1 Table). The mean coefficient of variation, skewness and kurtosis for the diameter of lesions as the phenotype values among the whole association panel are shown in Table 1 and Fig 1. The variation was observed, varying from 0.5 cm to 17.67 cm with an average value of 5.51 cm based on the data among the five tested time points. The coefficients of variation of the association population under different time points ranged from 29.27% to 39.04%. No significant skewness or kurtosis of the association panel was observed, which indicated that this association panel was suitable for subsequent GWAS analysis.

**Table 1:** Statistical analysis and variation for partial resistance to sclerotinia stem rot (SSR) among the association panel based on SPSS 19.0 software.

Time Point	Min(cm)	Max(cm)	Mean(cm)	CV (%)	Skewness	Kurtosis
3d	0.50	2.37	1.13	31.23	0.08	-0.060
4d	0.54	6.52	3.01	39.04	0.08	-0.096
5d	1.03	10.36	5.11	36.82	0.09	-0.003
6d	1.93	16.67	8.33	33.01	0.09	-0.094
7d	2.02	17.67	9.96	29.27	-0.10	-0.039

### Sequencing and SNP genotyping

**Fig 2:**
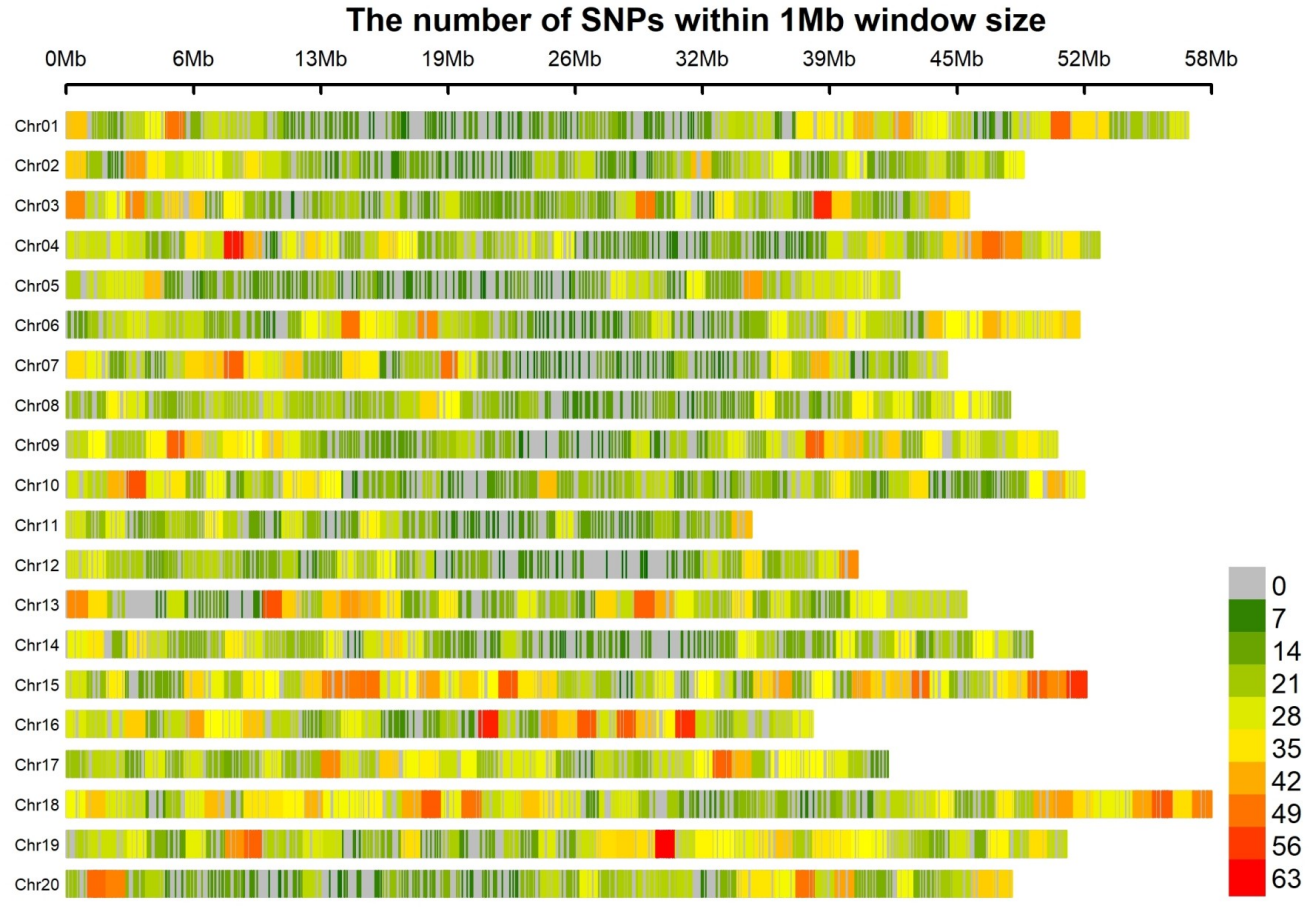
SNP density and distribution across the 20 soybean chromosomes.

The total DNA of the association panel containing 185 soybean accessions was partially sequenced through a specific-locus amplified fragment sequencing (SLAF-seq) approach. A total of 22,048 SNPs, distributed across all 20 chromosomes of the soybean genome, were identified with MAF > 5% and missing data < 3%. These developed SNPs spanned 917 Mbp, which could cover approximately 83.36% of the soybean genome (Fig 2). The number of SNPs was not even among the 20 chromosomes, with the highest and lowest number of markers being 1643 (in Chr.18) and 614 (in Chr.11), and the mean SNP per chromosome was 1102. The average marker density was approximately one SNP per 41.59 kb.

### Genome-wide association of SSR partial resistance

**Fig 3:**
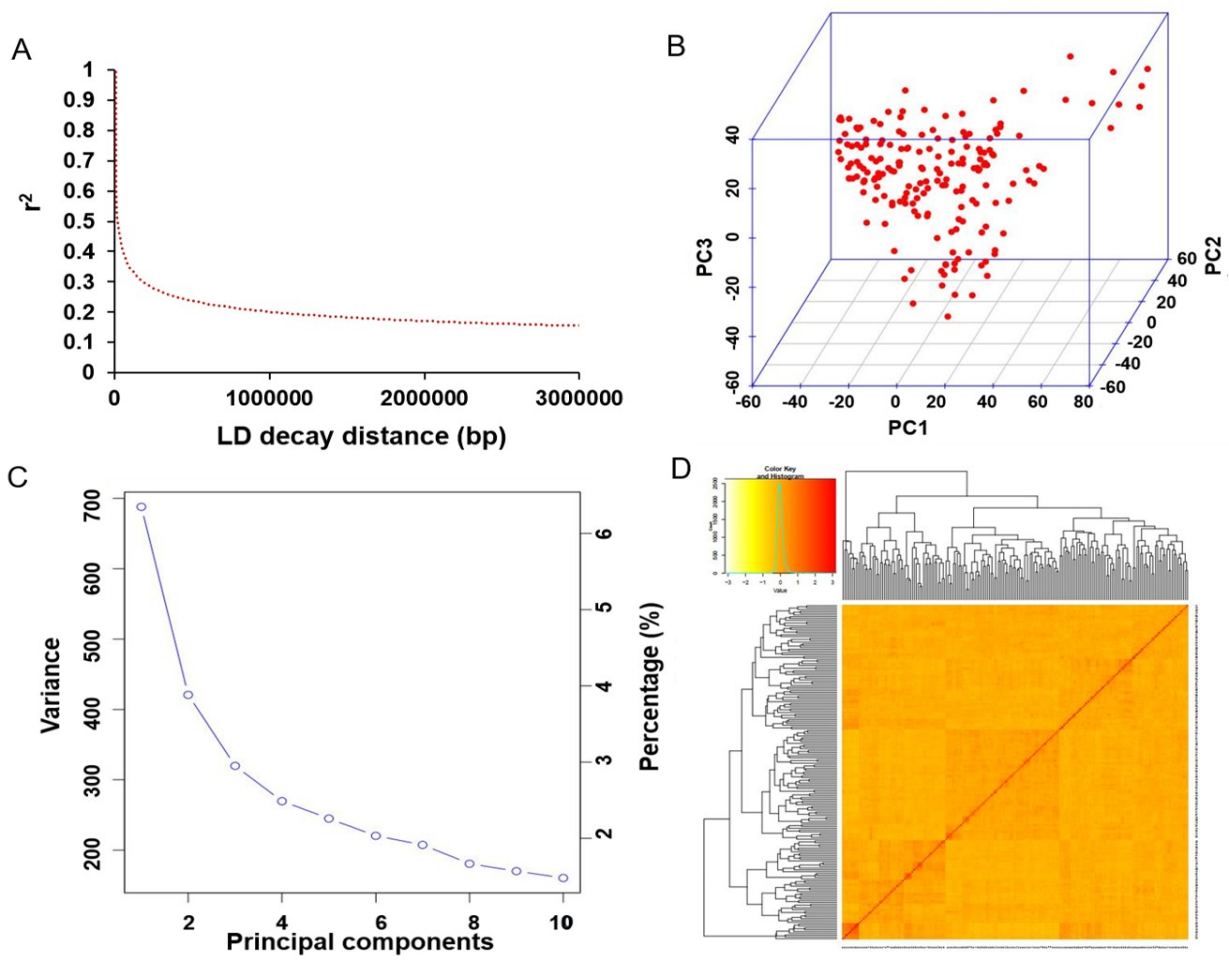
Genetic features of the mapping population. (A) The linkage disequilibrium (LD) decay of the genome-wide association study (GWAS) population. (B) The first three principal components of the more than 20,000 SNPs used in the GWAS. (C) Population structure of soybean germplasm collection as reflected by principal components. (D) A heatmap of the kinship matrix among the 185 soybean accessions.

The average distance of LD decay was analyzed to describe the mapping resolution for GWAS. The mean LD decay of the panel was estimated as 186.4 kb, when R^2^ decreased to half of its maximum value (Fig 3A). Principal component (PC) and kinship analyses were conducted to scan the population stratification of the association panel with the 22,048 SNP markers. The results showed that the first three PCs accounted for 13.18% of the overall genetic variations, and the inflection point occurred at PC3, which indicated that the first three PCs could dominate the population structure for the association mapping (Fig 3B and 3C). As shown in Fig 3D, a lower level of genetic relatedness was exhibited from the distribution of the pairwise relative kinship coefficients among the 185 tested accessions. Based on the CMLM model, seven major effects loci identified in at least two tested time points were associated with partial resistance to SSR, which were distributed on six chromosomes, including Chr.01, Chr.03, Chr.05, Chr.06, Chr.12 and Chr.18 (S1 Fig and Table 2). Among these loci, one QTN, Gm06:39027181 located on Chr.06, was found in the linked genomic region of the known QTL ‘Sclero7-1’ between the SSR markers of Sat_238 and Sat_312 [27], and the other six QTNs were considered as the novel loci for partial resistance to SSR (Table 2). Additionally, nine novel SNPs detected at least two time points were identified as the minor effects loci on the basis of mrMLM model, which were distributed on eight different soybean chromosomes (Table 3). One of the all the identified QTNs (Gm05:14834789 on Chr.05) was co-detected by both methods, giving more confidence that this locus might be significantly associated with soybean defense to SSR partial disease. The alleles effects of all the identified resistance QTNs were further analyzed, and the results showed that different alleles for each identified QTN could significantly affect SSR partial resistance among all the tested samples (Tables 2 and 3). Thus, these beneficial alleles would be valuable for MAS of soybean cultivars with excellent resistance to SSR.

**Table 2:** Peak SNPs and beneficial alleles associated with partial resistance to sclerotinia stem rot (SSR) identified by CMLM model.

SNP	Chr.	Position	Time	-log10 (P)	MAF	R^2^ (%)	QTN effects	Known QTL	Allele 1/Allele 2	Average diameter of lesions of accessions with allele 1/allele2(cm)	Average diameter of lesions of the population(cm)
Gm01:28271068	1	28271068	4d	3.08	0.06	6.66	-0.73		T/G	4.67/2.93	3.01
			6d	3.37	0.06	7.61	-1.81			11.92/8.12	8.33
Gm03:27991148	3	27991148	4d	3.68	0.09	8.18	-0.63		A/C	4.26/2.92	3.01
			5d	3.87	0.09	8.55	-1.05			7.03/4.95	5.11
Gm05:14834789	5	14834789	5d	3.61	0.17	7.89	-0.74		T/G	6.23/4.88	5.11
			6d	3.26	0.17	7.33	-0.99			9.89/8.01	8.33
			7d	4.28	0.17	10.68	-1.23			11.81/9.57	9.96
Gm06:39027181	6	39027181	5d	3.16	0.08	6.74	-0.94	Sclero7-1	T/G	6.69/4.97	5.11
			6d	3.19	0.08	7.16	-1.36			10.49/8.12	8.33
Gm12:7079865	12	7079865	6d	3.02	0.34	6.74	-0.76		C/T	8.85/7.34	8.33
			7d	3.14	0.34	7.77	-0.82			10.53/8.86	9.96
Gm12:36426007	12	36426007	5d	3.95	0.12	8.76	0.85		T/C	5.31/3.61	5.11
			6d	3.14	0.12	7.03	1.08			8.59/6.45	8.33
Gm18:949979	18	949979	5d	3.82	0.05	8.43	-1.54		T/G	7.26/4.94	5.11
			6d	3.37	0.05	7.61	-2.07			12.17/8.13	8.33
			7d	3.20	0.05	7.93	-2.12			13.62/9.75	9.96

**Table 3:** Peak SNPs and beneficial alleles associated with partial resistance to sclerotinia stem rot (SSR) identified by mrMLM model.

SNP	Chr.	Position	Time	LOD	-log10 (P)	MAF	R^2^(%)	QTN effects	Allele 1/Allele2	Average diameter of lesions of accessions with allele 1/allele 2(cm)	Average diameter of lesions of the population(cm)
Gm02:29817148	2	29817148	4d	7.78	8.68	0.07	5.81	-0.50	G/T	3.04/2.55	3.01
			5d	6.01	6.85	0.07	9.99	-1.19		5.16/4.40	5.11
			7d	5.04	5.83	0.07	5.20	-1.00		10.02/9.19	9.96
Gm05:14834789	5	14834789	5d	3.40	4.12	0.17	8.39	-0.74	G/T	5.18/4.80	5.11
			7d	3.59	4.32	0.17	3.39	-0.82		10.09/9.39	9.96
Gm07:33815183	7	33815183	6d	3.34	4.06	0.08	5.92	-1.23	G/T	8.50/7.53	8.33
			7d	6.56	7.41	0.08	4.49	-1.13		10.13/8.96	9.96
Gm07:529324	7	529324	4d	3.29	4.01	0.16	4.81	-0.32	C/T	3.02/2.99	3.01
			5d	5.29	6.09	0.16	8.43	-0.75		5.27/5.07	5.11
Gm09:5469571	9	5469571	4d	17.37	18.66	0.06	7.51	0.78	T/C	3.11/3.00	3.01
			6d	3.28	4.00	0.06	4.78	1.32		8.90/8.28	8.33
			7d	4.16	4.92	0.06	12.25	2.05		12.03/9.88	9.96
Gm15:47922300	15	47922300	6d	3.05	3.76	0.09	5.89	-1.08	T/A	9.24/8.26	8.33
			7d	5.33	6.14	0.09	3.52	-1.02		10.64/9.89	9.96
Gm16:21589672	16	21589672	3d	3.96	4.71	0.07	5.00	-0.11	G/T	1.16/1.12	1.13
			7d	6.29	7.13	0.07	4.73	-1.35		9.97/9.87	9.96
Gm18:6720660	18	6720660	4d	5.84	6.66	0.07	5.17	0.44	A/C	3.30/2.98	3.01
			5d	5.82	6.65	0.07	6.37	0.92		5.44/5.06	5.11
Gm19:36122367	19	36122367	6d	3.36	4.08	0.05	4.84	-1.37	C/A	8.44/6.99	8.33
			7d	6.78	7.63	0.05	4.11	1.16		10.06/9.70	9.96

### Prediction of candidate genes conferring partial resistance to SSR

Genes located within the 200-kb genomic region of each peak SNP identified by CMLM and mrMLM were considered as candidates, and finally, 71 major and 85 minor effects genes were found, respectively (S2 and S3 Tables). Some of these detected major genes have been identified to be of key importance in plant disease-related responses [28–32]. The research of Jennifer Colburn-Clifford and Caitilyn Allen has indicated that a cbb3-type cytochrome c oxidase could contribute to *Ralstonia solanacearum* race 3 biovar 2 (R3bv2) resistance in tomato [28]. In the present study, *Glyma*.*03G094400* (located 84.80 kb near Gm03:27991148 on Chr.03) and *Glyma*.*12G086600* (located 76.49 kb near Gm12:7079865 on Chr.12), containing the same functional domain mentioned above, might be regarded as important factors in SSR resistance to some extent. According to recent studies, Aubert et al [29]. and Gunupuru et al [30]. have reported that a cytochrome P450 gene from the subfamily CYP72A and CYP94C, respectively, conferred resistance to mycotoxin deoxynivalenol (DON) from wheat and *Botrytis cinerea* from *Arabidopsis*. These two genes had domains identical to *Glyma*.*06G238500* and *Glyma*.*12G087200*, located near Gm06:39027181 on Chr.06 and Gm12:7079865 on Chr.12. Additionally, *Glyma*.*01G093400* (located 47.84 kb near Gm01:28271068 on Chr.01), a basic helix-loop-helix (bHLH) DNA-binding superfamily protein, was of particular importance in affecting the regulation of disease resistance in plants [31]. *Glyma*.*18G013200* (located 16.91 kb near Gm18:949979 on Chr.18), a member of mitogen-activated protein kinase phosphatase 1, was shown to be a significant repressor in salicylic acid synthesis and made strong contributions to defense responses [32]. Moreover, three candidate genes with minor effects (*Glyma*.*07G007800*, *Glyma*.*07G007900* and *Glyma*.*09G059700*), contained the common disease resistance proteins, including disease resistance protein (TIR-NBS-LRR class) and pathogenesis related homeodomain protein of each own, were detected and recognized as potential genes in soybean SSR responses.

**Fig 4:**
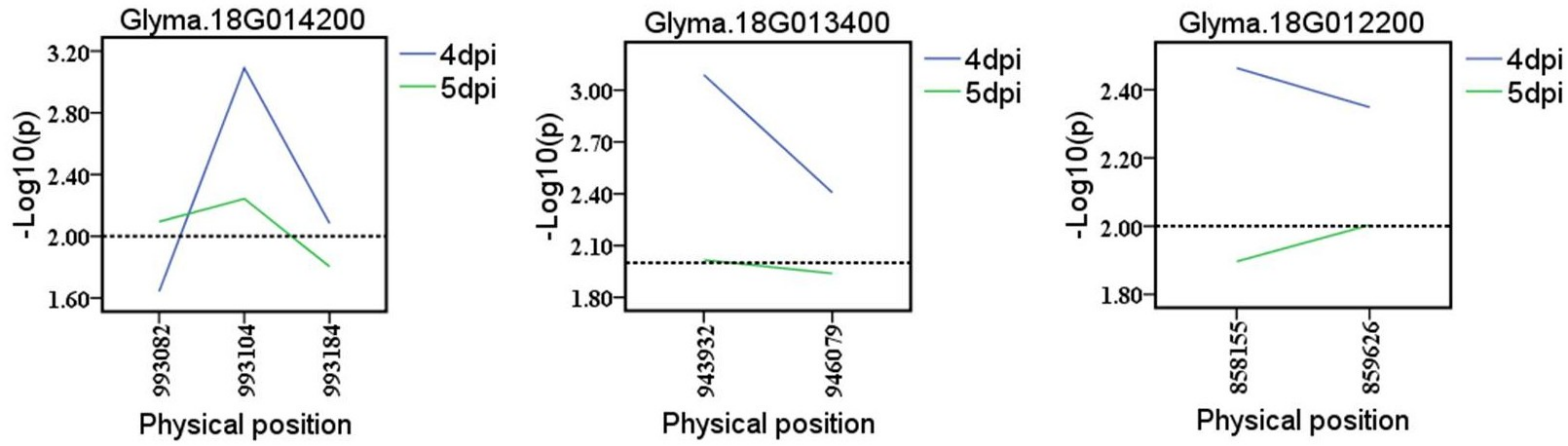
Candidate gene-based association. Gene-based association analysis of major candidate genes with SNPs that were significantly correlated to partial resistance to sclerotinia stem rot (SSR). The horizontal line indicated that the threshold was set to 2.0.

**Fig 5:**
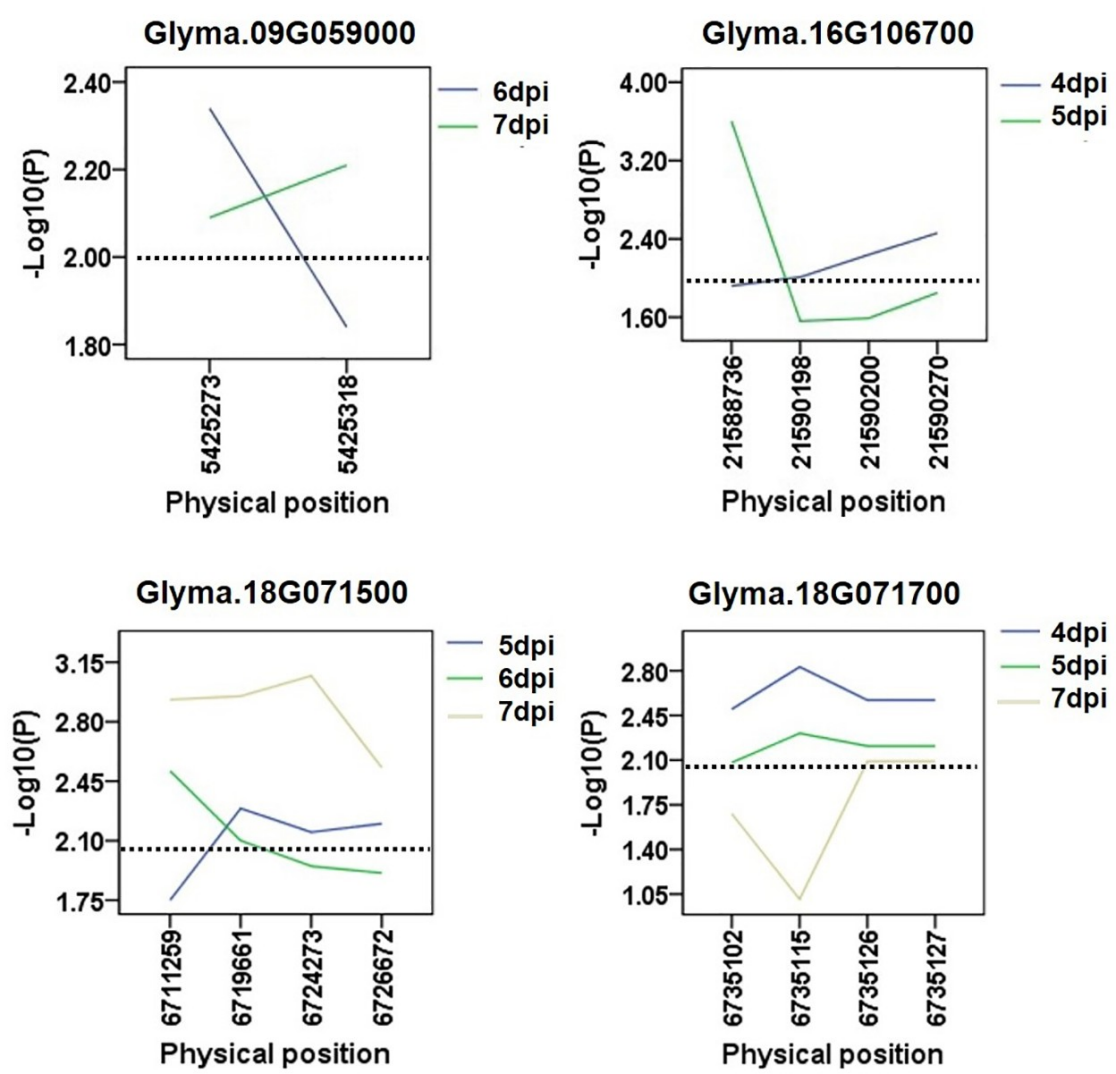
Candidate gene-based association. Gene-based association analysis of minor candidate genes with SNPs that were significantly correlated to partial resistance to sclerotinia stem rot (SSR). The horizontal line indicated that the threshold was set to 2.0.

**Fig 6:**
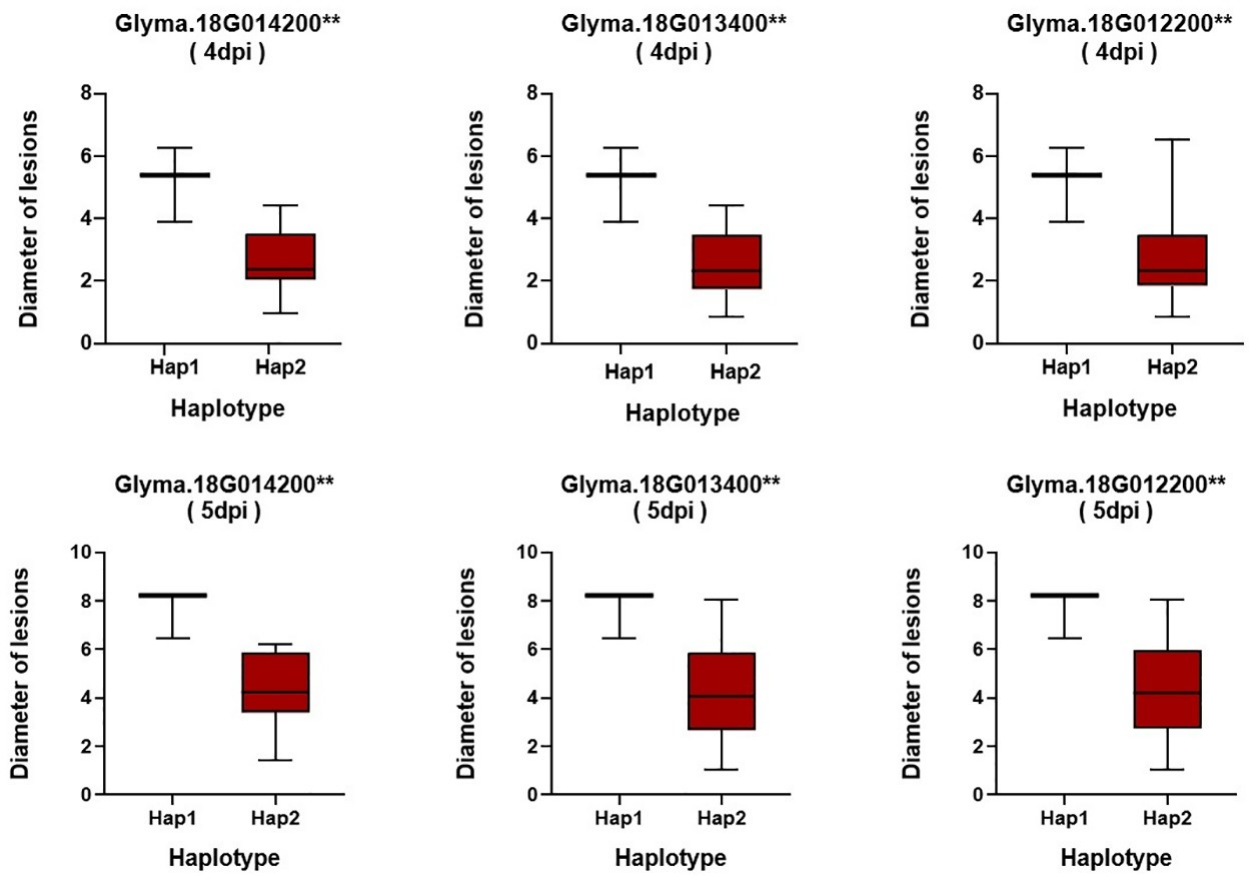
Haplotype analysis of major candidate genes with variations related to partial resistance to sclerotinia stem rot (SSR). The ** value suggested significance of ANOVA at *p* < 0.01, respectively.

**Fig 7:**
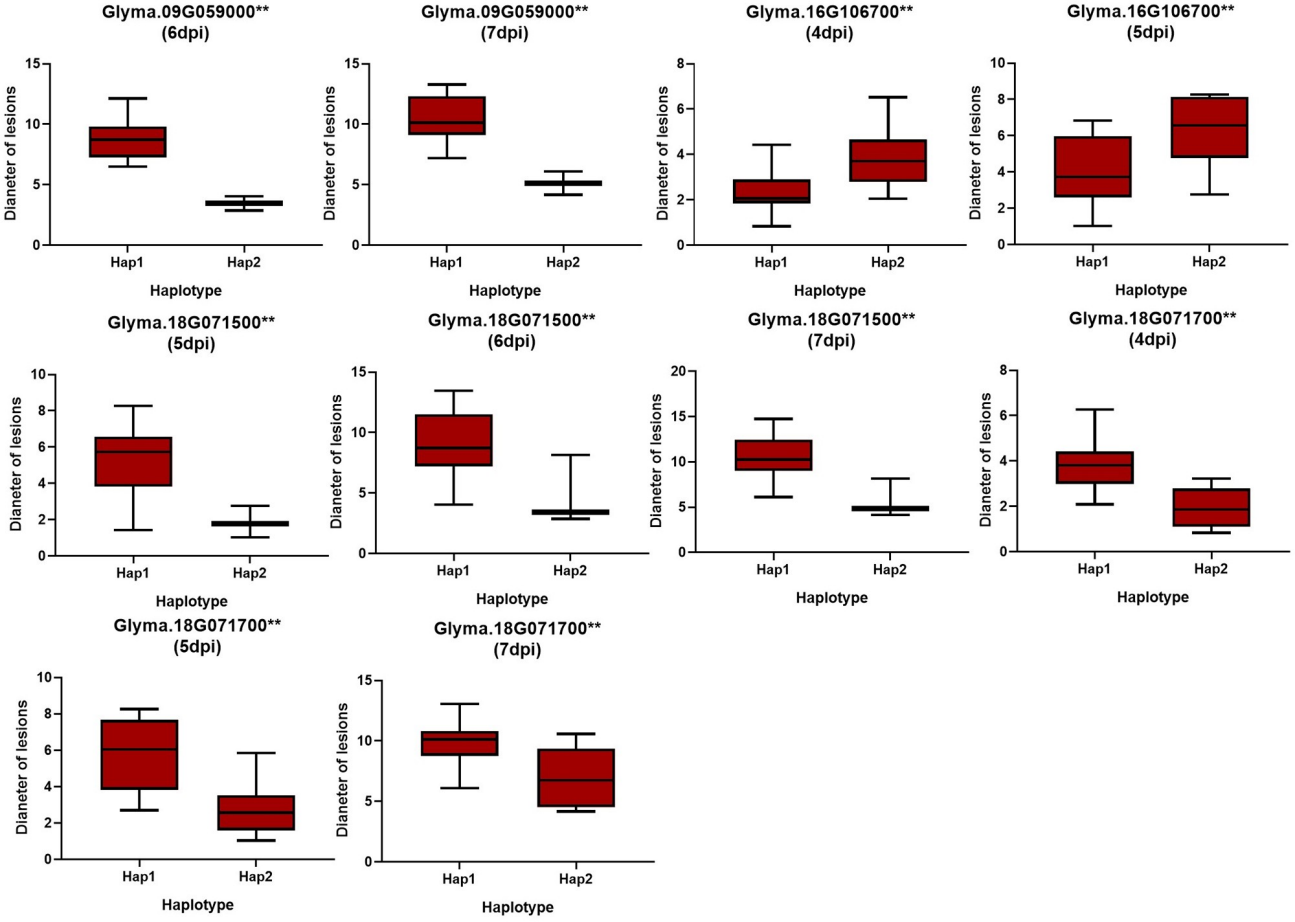
Haplotype analysis of minor candidate genes with variations related to partial resistance to sclerotinia stem rot (SSR). The ** value suggested significance of ANOVA at *p* < 0.01.

To further determine the possibilities of the candidate genes affecting the partial resistance to SSR, a gene-based association was conducted by the CMLM method among 13 resistant and 13 susceptible soybean lines (S2 Fig). Finally, a total of 87 SNPs from 71 major candidate genes and 102 SNPs from 85 minor candidate genes were identified on the basis of genome resequencing (MAF > 0.05), respectively. Of them, six haplotypes obtained from three genes with major effects (*Glyma*.*18G012200*, *Glyma*.*18G013400* and *Glyma*.*18G014200* located near Gm18:949979 on Chr.18) out of 71 candidates were found to be significantly associated with partial resistance to SSR (Figs 4 and 6 and S4 Table). Among these three genes, *Glyma*.*18G012200*, encoding a pectate lyase protein, has been reported as an essential effector in *Colletotrichum lindemuthianum* (one of the most common and important genera of phytopathogenic fungi) in plants [33]. The other two genes (*Glyma*.*18G013400* and *Glyma*.*18G014200*) could be considered novel genes conferring partial resistance to SSR. Additionally, eight haplotypes derived from four novel genes with minor effects (*Glyma*.*09G059000*, *Glyma*.*16G106700*, *Glyma*.*18G071500* and *Glyma*.*18G071700*) out of 85 candidates were identified in relation to SSR partial resistance (Figs 5 and 7 and S5 Table). Given the results of the allelic effects analysis on the two alleles of each peak SNP, the partial resistance to SSR of soybean accessions with one allele was noticeable, unlike the other alleles among these total seven candidates (Figs 6 and 7). Therefore, these beneficial alleles from the candidate genes, exhibiting excellent partial resistance to SSR, may be interesting and helpful for studies employing MAS in soybean. Moreover, the gene expression levels under biotic stresses recorded on BAR database were applied in analyzing the potential resistances in fungal diseases of the detected major or minor candidate genes (S3 and S4 Figs). Among them, *ATMG01360*, *AT3G55270*, *AT4G40080* and *AT3G13330*, which were homologous to *Glyma*.*03G094400*, *Glyma*.*18G013200 Glyma*.*18G014200* and *Glyma*.*18G071500*, exhibited a higher expression after inoculating *Botrytis cinereal* and *Phytophthora infestans* (close relative to *Sclerotinia sclerotiorum*) than the mock treatments, implying that these four candidates might act as a positive regulator in controlling these two fungal diseases. On the contrary, the expressions of *AT1G67750*, *AT4G30020* and *AT1G31410* homologous to *Glyma*.*18G012200*, *Glyma*.*09G059000* and *Glyma*.*16G106700*, were reduced after inoculating *Botrytis cinereal* and *Phytophthora infestans* than that in the blank control group, thus, *Glyma*.*18G012200*, *Glyma*.*09G059000* and *Glyma*.*16G106700* were likely as negative regulatory factors in the disease resistance. Besides, *AT4G21105*, *AT3G26370*, *AT1G72890* and *AT5G23810* (homologous to *Glyma*.*12G086600*, *Glyma*.*18G013400*, *Glyma*.*07G007900* and *Glyma*.*18G071700*) were responded to *Phytophthora infestans* with the up-regulated or down-regulated gene expression after treatments, and *AT5G17680* (homologous to *Glyma*.*07G007800*) was responded to *Botrytis cinereal* with the highly upregulated expression.

## Discussion

Sclerotinia stem rot (SSR) is one of the most devastating fungal phytopathogens, causing severe annual yield losses of soybean worldwide. As a heritable complex quantitative trait, SSR is controlled by multiple genes with major or minor effects [6] and is easily affected by environment and genotype through environment interactions [5]. Although no related research on complete resistance to SSR has been reported in soybean, partial resistance was still an alternative in effectively controlling SSR to a large extent [5]. Breeding resistant cultivars with durable resistance genes/QTLs was still the most economical and long-lasting means of managing this disease. To date, more than a hundred QTLs on partial resistance to SSR have been reported based on linkage mapping strategy and the bi-parent population (www.soybase.org). However, few of these QTLs could be widely utilized in MAS for partial resistance to soybean SSR. In the present study, a group of 185 soybean accessions, including landraces or elite cultivars mainly collected from China, was used to examine partial resistance to soybean SSR *via* GWAS based on the high-throughout SNPs. The results showed that some of the tested samples exhibited a higher level of partial resistance to SSR within a shorter lesion diameter during the dynamic stages (Fig 1 and S1 Table). These accessions with partial resistance might be valuable resources for soybean SSR resistance for breeding new varieties.

In the present study, the data from the consecutive five days after inoculating detached leaves for 48 h were not only used to screen resistant or susceptible soybean lines, but also applied to observe the differences of SSR spreading speed, and thereby, to find the SSR-related resistance loci and genes. Finally, a total of 60 QTNs with major effects (under 3dpi, 7 QTNs; under 4dpi, 22 QTNs; under 5dpi, 16 QTNs; under 6dpi, 9 QTNs; under 7dpi, 6 QTNs) were positioned among the five tested time points associated with partial resistance to soybean SSR by GWAS based on the CMLM model (S1 Fig). Further, seven major QTNs, which were stably detected under at least two time points, were considered to be the more significant ones. Among them, Gm06:39027181 on Chr.06, identified under ‘5d and 6d’, was found to overlap with the known resistance QTL, known as ‘Sclero7-1’, detected between the SSR markers ‘Sat_238’ and ‘Satt_312’ by using the cross population with 180 F_4_ RILs derived from a cross between Maple Donovan (a partially resistant cultivar) and OAC Bayfield (a susceptible cultivar) [27]. The other six identified QTNs, including Gm01:28271068 under 4dpi and 6dpi, Gm03:27991148 under 4dpi and 5dpi, Gm 05:14834789 under 5dpi, 6dpi and 7dpi, Gm12:7079865 under 6dpi and 7dpi, Gm12:36426007 under 5dpi and 6dpi, and Gm18:949979 under 5dpi, 6dpi and 7dpi, were marked as novel loci with strong possibilities in controlling partial resistance of soybean SSR. Additionally, for effectively detecting the loci with minor effects, a GWAS based on the mrMLM model was conducted, and nine novel QTNs detected at least two time points were identified and regarded as the minor effects loci associated with SSR partial resistance. Gm05:14834789 on Chr.05 was co-located by both models, giving more possibilities that this locus might be a stable one in association with the defense to soybean SSR partial disease.

To date, few genes associated with partial resistance of SSR have been found or cloned. Zhao et al. identified four genes that were involved in disease response or the anthocyanin biosynthesis pathway using GWAS and linkage maps [5]. GWAS has been an effective method to identify candidate genes for SSR partial resistance, especially with a relatively lower LD block. In this study, a total of 71 and 85 potential candidate genes with major or minor effects were selected on the basis of the seven and nine association signals *via* CMLM and mrMLM, respectively. Of these candidate genes, *Glyma*.*01G093400* (located near Gm01:28271068 on Chr.01), *Glyma*.*03G094400* (located near Gm03:27991148 on Chr.03), *Glyma*.*06G238500* (located near Gm06:39027181 on Chr.06), *Glyma*.*12G086600* and *Glyma*.*12G087200* (located near Gm12:7079865 on Chr.12), and *Glyma*.*18G013200* (located near Gm18:949979 on Chr.18) have been demonstrated to be typical of factors participating in plant disease-related responses [28–32]. Furthermore, combination with the data from the *Arabidopsis* BAR database, we found six major effects genes (*Glyma*.*03G094400*, *Glyma*.*12G086600*, *Glyma*.*18G012200*, *Glyma*.*18G013200*, *Glyma*.*18G013400* and *Glyma*.*18G014200*) and six minor effects genes (*Glyma*.*07G007800*, *Glyma*.*07G007900*, *Glyma*.*09G059000*, *Glyma*.*16G106700 Glyma*.*18G071500* and *Glyma*.*18G071700*) might responded to the fungal stress. To further determine the possibilities of the candidate genes affecting the partial resistance to SSR, a gene-based association was performed. Finally, three major genes with six beneficial haplotypes (*Glyma*.*18G012200*, 2 SNPs; *Glyma*.*18G013400*, 2 SNPs; and *Glyma*.*18G014200*, 2 SNPs) and four minor genes with eight beneficial haplotypes (*Glyma*.*09G059000*, 2 SNPs; *Glyma*.*16G106700*, 2 SNPs *Glyma*.*18G071500*, 2 SNPs and *Glyma*.*18G071700*, 2 SNPs) were screened. Of these genes, *Glyma*.*18G012200*, belonging to the pectate lyase superfamily protein, has been identified as a key effector in controlling *Colletotrichum lindemuthianum*, recognized as one of the most common and important genera of phytopathogenic fungi, in plants [33]. The other six genes, each containing two allelic variations occurring in major regions, may also have potential for managing partial resistance to soybean SSR. Moreover, a possibility of interaction between the *Glyma*.*09G059000* and *Glyma*.*18G071500* was existed recorded on the STRING database (https://string-db.org/), predicting that these two genes might work together in soybean SSR resistance. Further studies on the definite functions and specific mechanisms of these candidates will be conducted and further analyzed.

## Supporting information

S1 Fig Manhattan plot of association mapping of partial resistance to sclerotinia stem rot (SSR) *via* CMLM model. ‘A, B, C, D, E’ represented the tested time points of ‘3d, 4d, 5d, 6d and 7d’.
(JPG)

S2 Fig Extreme phenotypes of representative accessions among the 26 selected for genome re-sequencing. (JPG)

S3 Fig Expressions of six *Arabidopsis* genes under fungus stress, which were homologous to the major candidate genes for soybean sclerotinia stem rot (SSR). (JPG)

S4 Fig Expressions of six *Arabidopsis* genes under fungus stress, which were homologous to the minor candidate genes for soybean sclerotinia stem rot (SSR). (JPG)

S1 Table List of 185 soybean accessions with phenotype values. (XLSX)

S2 Table Candidate genes in the 200 kb flanking region of peak SNPs detected by CMLM model. (XLSX)

S3 Table Candidate genes in the 200 kb flanking region of peak SNPs detected by mrMLM model. (XLSX)

S4 Table Haplotype analysis of candidate genes with major effects. (XLSX)

S5 Table Haplotype analysis of candidate genes with minor effects. (XLSX)
