# Supplementary figures and images for: Genome-wide association study of partial resistance to sclerotinia stem rot of cultivated soybean based on the detached leaf method
Source: PLoS One. 2020 May 18;15(5):e0233366. doi: 10.1371/journal.pone.0233366 (PMC7233537; doi:10.1371/journal.pone.0233366)

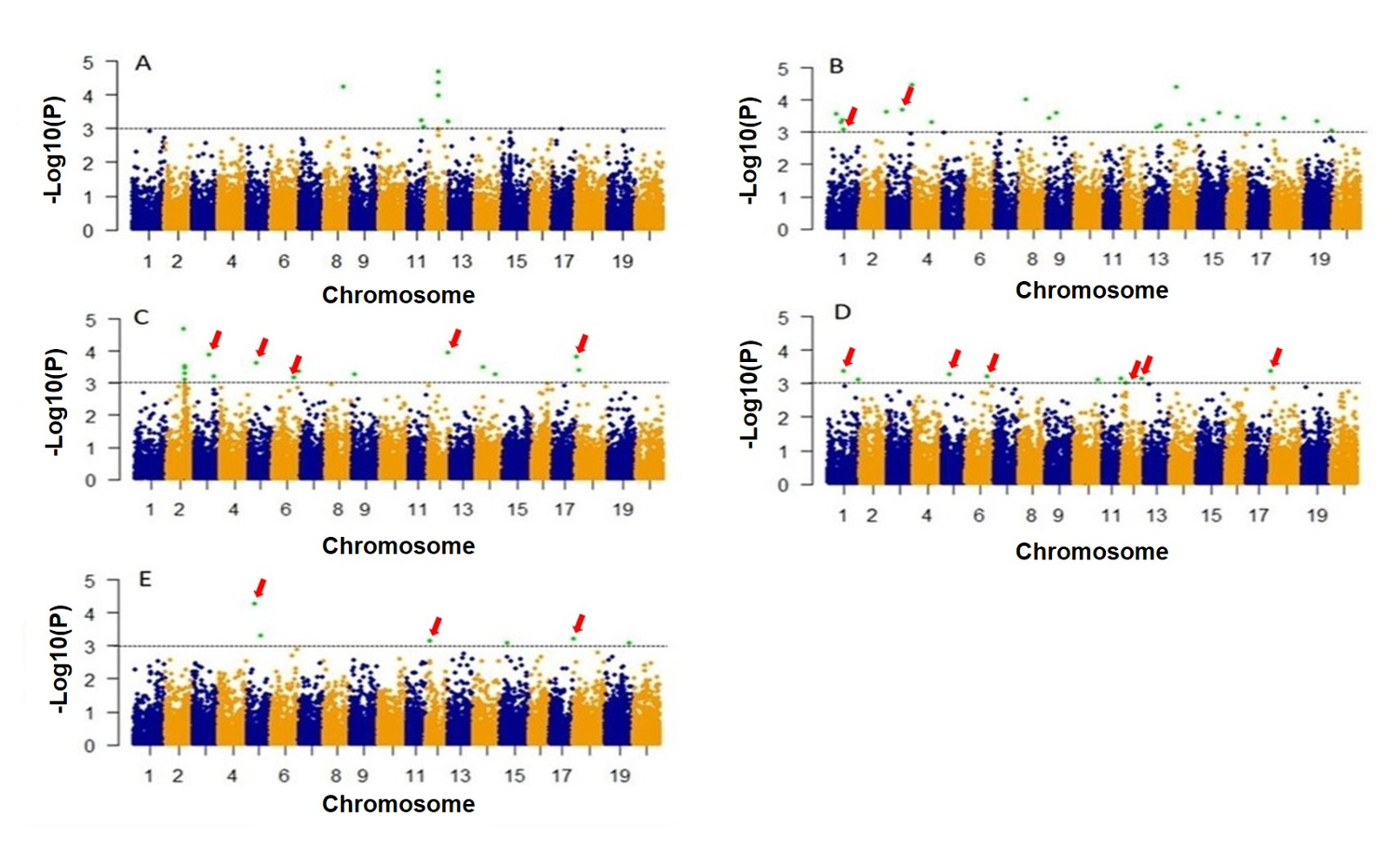

Supplement: S1 Fig — ‘A, B, C, D, E’ represented the tested time points of ‘3d, 4d, 5d, 6d and 7d’. (JPG) [file pone.0233366.s001.jpg]

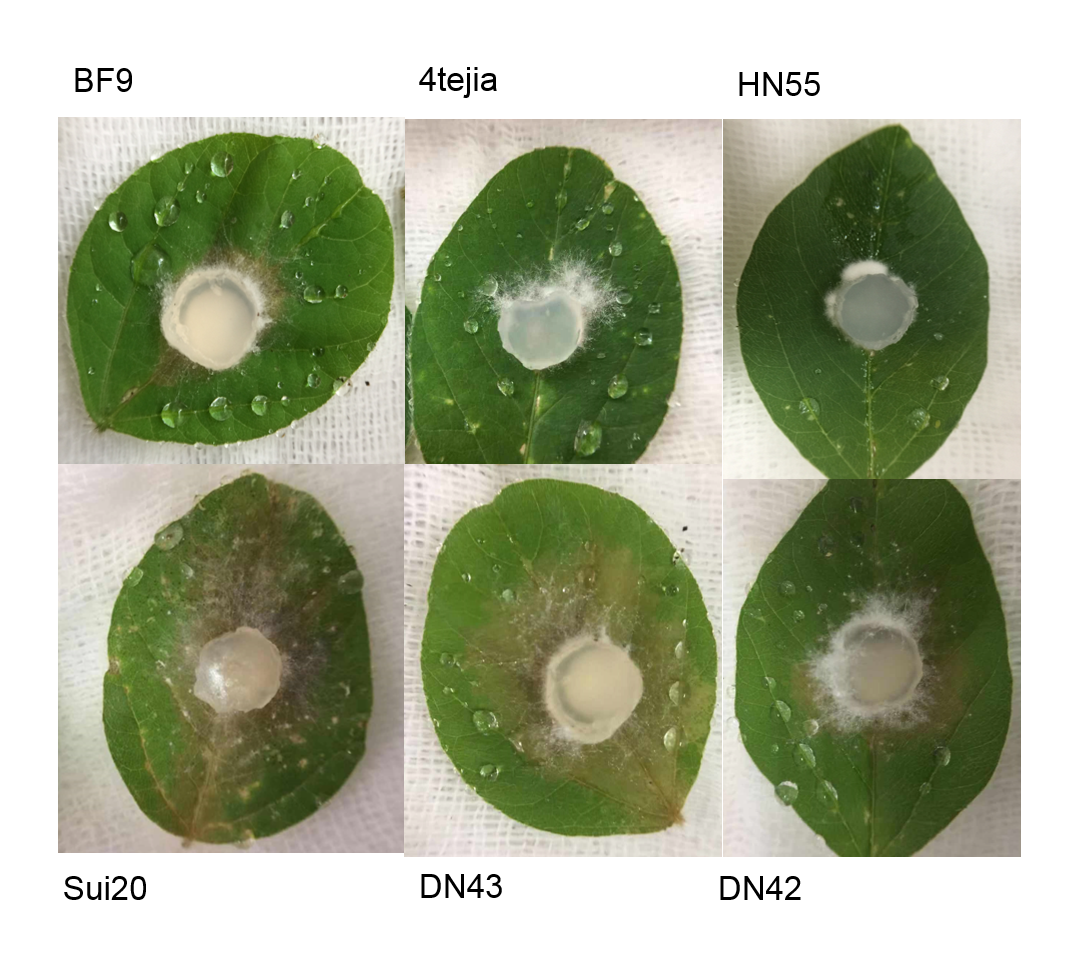

Supplement: S2 Fig — (JPG) [file pone.0233366.s002.jpg]

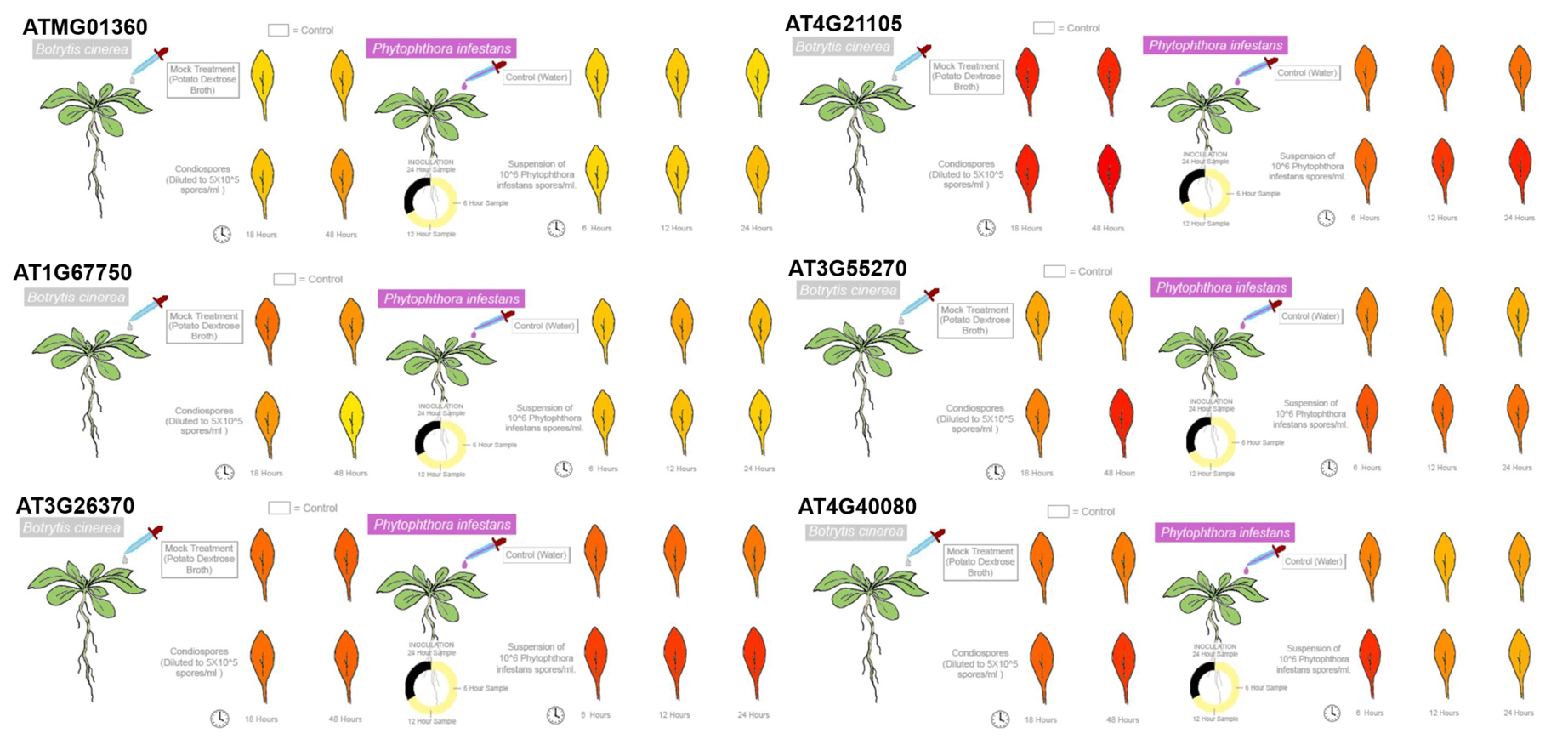

Supplement: S3 Fig — (JPG) [file pone.0233366.s003.jpg]

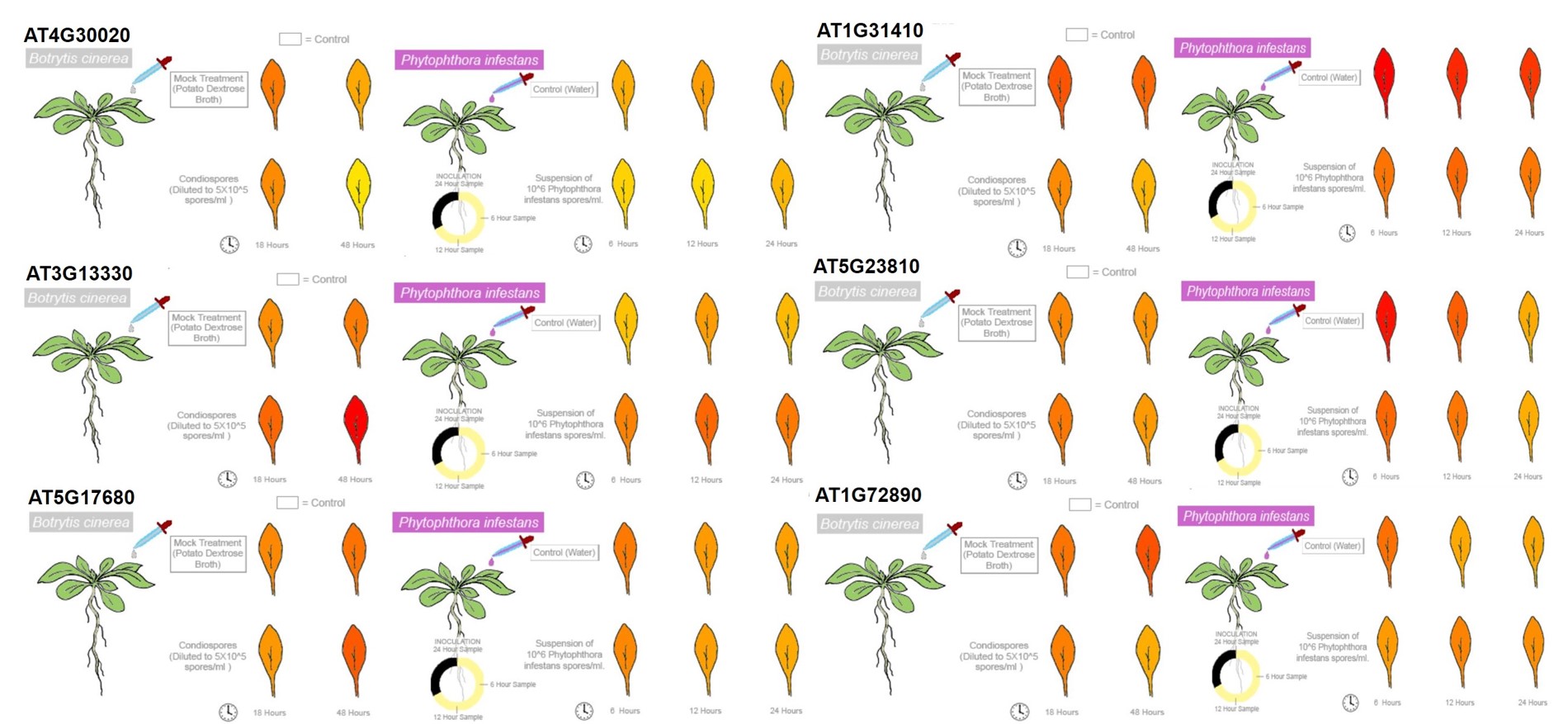

Supplement: S4 Fig — (JPG) [file pone.0233366.s004.jpg]
